# The relationship between socioeconomic status and risky drinking in Denmark: a cross-sectional general population study

**DOI:** 10.1186/s12889-018-5481-y

**Published:** 2018-06-15

**Authors:** Abdu Kedir Seid, Kim Bloomfield, Morten Hesse

**Affiliations:** 1 Copenhagen, Denmark; 2 Centre for Alcohol and Drug research, Aarhus University, Bartholins Allé 10, Copenhagen, 8000 Denmark

**Keywords:** Socioeconomic status, Education, Income, Employment, Risky single occasion drinking, Denmark

## Abstract

**Background:**

Socioeconomic status (SES) is regarded as consisting of education, income and employment. However, the relationship of these three components to alcohol use behaviours, such as risky single occasion drinking (RSOD) is unclear. The aim of the present paper is to specify how the three SES components relate to RSOD in a cross-sectional survey sample of the Danish general population.

**Method:**

Data from a 2011 Danish national representative survey (*n* = 3600) was analysed by multiple logistic regression to assess the influence of three dimensions of individual SES (education, income, employment) on RSOD.

**Results:**

Components of SES were not found to be significantly associated with RSOD independently nor in combination.

**Conclusion:**

In the Danish context, SES was not associated with RSOD.

## Background

A major aim of social epidemiology is the study of the relationship between socioeconomic status (SES) and health outcomes in populations, and to date it is well known that in almost every country, more people of lower SES report and experience poorer health than those of higher SES; this is especially true in the developed world [1].

SES generally refers to the social standing, rank or class of an individual or group in society [2]. It is often operationalized and measured as a combination of education, income and occupation [3]. SES is believed to influence health through a combination of increased knowledge, access to financial resources, and access to social support, as is purported in the “fundamental cause theory” of Link and Phelan [4]. However, the specific ways in which SES may influence health are still not fully understood.

An argument can be made that education should be the key component of SES in relation to health, because education enables people to understand health information and make healthier choices [5]. Furthermore, it can influence behaviour and attitudes, which in turn can affect relationships with healthcare providers [6]. Although some studies have shown that higher education have a positive effect on health and health behaviour [7–9], others argue that it is not education per se, but rather the other components of SES which are associated with education that influence health [7]. For example, studies have shown that better educated individuals tend to have better jobs with higher incomes, which allow them to invest more in health care, and to afford a healthier lifestyle (such as better access to healthier foods, and membership fees for athletic clubs, etc.). Additionally, higher education could also afford access to employment in healthier work environments as well as the means to avoid work-related stress [9–12].

### SES and alcohol use

With regard to the specific health behaviour of alcohol use, research has shown that individual SES (measured by all components: education, income, and occupation) is associated in various ways with risky alcohol consumption [13–15]. In the same manner that individual health risk factors such as smoking and sedentary lifestyles are more prevalent among lower SES groups [14, 16, 17], those with low SES are also more likely to suffer from alcohol-related morbidity and mortality [18–20]. However, regarding drinking patterns, people with low income have been found to be more likely to be either abstainers or heavy drinkers, and less likely to be moderate drinkers [21] or to drink frequently [22]. Thus, research has shown that the social gradient does not always follow in the same direction. Some findings from higher income Western countries indicate that those with higher education and/or income can drink at harmful levels (e.g. [23]).

One way to further investigate the link between SES and drinking is by assessing the different potential relationships from the various components of SES to drinking patterns. So far, research that attempts to do this is rare, but one such study that used a variety of data sets from UK and USA found a strong mediation effect of income on the relationship between education and health behaviours [24]. Specifically, income was found to reduce the effect of education on current smoking by 26% and on heavy drinking by 12%, indicating that income mediated the effect of education on smoking and drinking.

In general, people aged 25 years or older have completed their education, thus this adult age group is an appropriate population in which to study the effects of education as a stable component of SES [3, 24]. However, the links between education and employment status are more complicated, as type of employment may influence health through the prestige associated with the type of job, which, in turn, may again vary between countries and may have changed over time [3]. Education is considered in this study to be an independent component of adult SES (i.e., influencing income or job status), and income or job status are then tested as potential mediators that more directly influence drinking behaviour.

To shed new light on the links between SES and alcohol indicators, the present study specifically addresses the following research questions: (a) Is education associated with risky single occasion drinking (RSOD) in Denmark? (b) Does income or employment mediate the effects of education on RSOD? We analyse general population survey data from Denmark to address this gap in the alcohol research literature. We examine a recognised indicator of possible problematic drinking, risky single occasion drinking (RSOD), as our outcome variable [25].

## Methods

### Design and setting

**Fig. 1:**
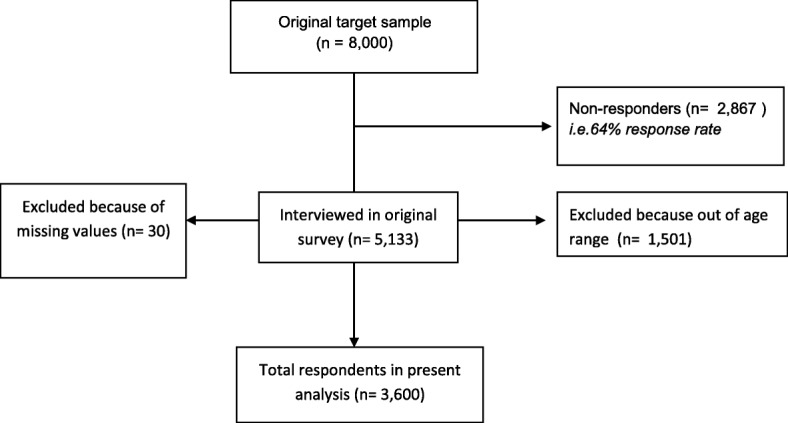
Flow chart describing selection of the survey data for present analysis

Data came from the 2011 national survey of alcohol and drugs conducted by Statistics Denmark for the Centre for Alcohol and Drug Research of Aarhus University. A representative sample of 8000 persons between the ages of 15–79 years old was randomly drawn from the central person registration (CPR) numbers. Upon birth or immigration to the country, each resident is assigned a unique registration number, which is used for official record keeping. Potential respondents (i.e., the 8000 potential cases) were invited by postal letter to complete a web questionnaire during September and October 2011. Telephone interviews were conducted with those individuals who had not responded after two reminders. The interviewed sample consisted of 5133 respondents representing a response rate of 64%. The Danish Data Protection Agency approved the survey and all confidentiality and privacy requirements were met. Registry data from Statistics Denmark were used to include information about respondents’ years of schooling and disposable income. The survey’s age range was restricted to correspond roughly to the end of tertiary education and at the high-end age for the start of retirement (26–67 years) [24]. This resulted in a final sample of 3600 individuals for the analyses (see Fig. 1).

### Drinking variables

To measure alcohol consumption respondents were first asked whether they had drunk any alcohol in the past 12 months or not. In a separate question, current drinkers were asked about their frequency of RSOD during the previous 12 months. RSOD was defined as drinking five or more units of alcohol (equivalent to 60 g of alcohol or more) on a single occasion. Respondents had a choice of different frequencies, which we coded as one if respondents reported any RSOD at least once per month in the last 12 months or otherwise as zero.

### Socioeconomic status

The main independent variable of interest was SES, specifically its three components education, income, and employment status. As a measure of education, we used years of schooling. We measured income using personal disposable income, which is the summation of pre-tax income and imputed rent minus interest expenses, tax, and paid alimonies. In the analysis, its logarithmic form was designed to reduce skewness. Data on education and personal disposable income were obtained from Statistics Denmark and merged with the survey data. The survey asked respondents to provide their employment status by choosing from given categories in the questionnaire. For the analyses, we coded one when respondents were employed and zero otherwise.

### Demographic variables

The sociodemographic variables used in the analysis include age groupings (26-35, 36-45, 46-55, and 56 years and older), gender and civil status (in a relationship i.e. married and/or living with a partner, not in a relationship i.e. divorced/separated/widowed, and never married). We also created a binary indicator of whether the respondent was living with children under the age of 18 years. Our indicator of religiosity equals one if the respondent reported attending worship or religious ceremonies more than four times in the last 12 months. Finally, we included area of residence (coded as capital, other large cities, rural).

### Statistical analyses

The pairwise correlation between RSOD and each covariate was examined using the Kruskal-Wallis equality-of-populations rank test and Spearman’s correlation test. To examine the association between components of SES and RSOD, we used multiple logistic regression. To avoid small cell frequencies, we dichotomized RSOD (at least monthly RSOD in the past year versus all other). In the analyses, we followed three steps. In the first step, the relationship between education and RSOD was analysed without including the other components of SES while controlling sociodemographic variables. In the second step, we added income to the model. In the third step, we added employment status.

In all analyses, we controlled age, gender, marital status, living with children under the age of 18, religiosity, and area of residence, as these variables appear from the literature to be key sociodemographic factors affecting drinking. We used weighted data, using weights, which were created by Statistics Denmark and are based on national distributions of age, sex, family structure, education, income, and country of origin. We conducted all analyses with STATA 14.0 software [26].

## Results

Table 1 presents descriptively the study variables. Women were slightly overrepresented in the final study sample. The majority of respondents reported being in the age group 45–64, being employed, in a relationship, and living in cities. Overall, over a third of male respondents in the sample reported RSOD at least monthly in the last 12 months. The proportion of those engaging in RSOD was higher for men than women (*p* < 0.001; data not shown). The Spearman rank correlation shows that RSOD was positively correlated with age, education, income, employment, religiosity, and residence areas. Furthermore, education was positively correlated with income and employment (*p* < 0.001; data not shown).

**Table 1:** Study sample characteristics (n, %) unweighted data, *n* = 3600

Study variables	Total	RSOD at least once monthly (%)	*p*-values^c^
RSOD^a^			
Never	1079 (29.7)		
Less than monthly	1628 (44.9)		
1–3 times per month	676 (18.6)		
At least 1–3 times per week	247 (6.8)		
Female	1958 (54.3)	16.2	< 0.001
Male		36.6	
Age			< 0.001
26–35	621 (17.2)	35.1	
36–45	907 (25.2)	23.9	
46–55	1040 (28.8)	25.6	
56+	1036 (28.8)	21.0	
Education, mean years of schooling (SD)^b^	14.3 (2.94)		0.635
Log income, mean (SD)	5.5 (0.21)		< 0.001
Employment status			
Employed	2534 (70.3)	27.1	< 0.001
Student/pupil	105 (2.9)	41.9	
Unemployed	108 (3.0)	26.2	
Pensioner	595 (16.5)	17.5	
Other including homemakers	266 (7.3)	21.2	
Civil status			< 0.001
In relationship	2927 (81.2)	24.0	
Not in a relationship	322 (9.0)	26.7	
Single	354 (9.8)	36.5	
Living with children (< 18 yr.)	1499 (41.7)	21.9	< 0.001
Religiosity^b^	597 (16.6)	19.4	< 0.001
Residence area			< 0.001
Capital	699 (19.4)	32.0	
Rural	1519 (42.2)	21.6	
Other cities	1384 (38.4)	26.0	

Note:^a^ RSOD i.e. risky single occasion drinking was defined as drinking 5+ standard drinks on one occasion and we dichotomised it as one reporting at least once in a month in the last 12 months and zero otherwise

^b^attending religious ceremonies more than 4 times in the previous year, *SD* standard deviation

^c^Spearman’s rank test between RSOD and covariates

Table 2 reports the odds ratios for multiple logistic regressions in which we estimated three different models to investigate the direct impact of education on RSOD while controlling sociodemographic variables. The first column (Model 1) reports the odds ratios of education in years of schooling as the chosen component of SES, the second column shows Model 2, in which income was added to the model, and the third column shows model 3, in which employment status was added. The results show that none of the SES variables were associated with the probability of reporting RSOD in any of the models. All models showed that being a woman, older and religious, as well as living with children under 18, and living in rural areas were negatively associated with reporting RSOD.

**Table 2:** Multiple logistic regression results for RSOD regressed on SES and sociodemographic variables, odds ratios (OR) and 95% confidence intervals (CI) (*N* = 3600)

	Education + sociodemographics	Education, income + sociodemographics	Education, income, employment + sociodemographics
Variables	OR [95% C.I.]	OR [95% C.I.]	OR [95% C.I.]
Education	1.01 [0.98–1.04]	1.00 [0.97–1.03]	1.00 [0.97–1.04]
Income		1.10 [0.72–1.68]	0.91 [0.56–1.47]
Employed			1.23 [0.98–1.56]
Gender (ref. male)	0.33 [0.28–0.40]***	0.33 [0.28–0.40]***	0.32 [0.28–0.40]***
Age (ref. 26–35)			
36–45	0.69 [0.54–0.89] **	0.68 [0.53–0.88] **	0.68 [0.53–0.88] **
46–55	0.65 [0.51–0.83]**	0.64 [0.50–0.83]**	0.65 [0.50–0.84]**
56+	0.41 [0.31–0.54]***	0.41 [0.31–0.55]***	0.44 [0.33–0.59]***
Civil status (ref. relationship)			
No relationship	1.33 [1.00–0.96]	1.31 [0.98–1.75]	1.32 [0.98–1.77]
Single	1.27 [0.96–1.68]	1.28 [0.96–1.70]	1.30 [0.97–1.72]
Religion	0.73 [0.58–0.93]*	0.78 [0.62–0.98]*	0.69 [0.54–0.88]**
Lived with child younger than 18	0.58 [0.47–0.72]***	0.62 [0.49–0.79]***	0.61 [0.49–0.75]***
Residence area (ref. capital)			
Other cities	0.78 [0.62–0.97]*	0.78 [0.62–0.98]*	0.78 [0.62–0.97]*
Rural	0.62 [0.49–0.78]***	0.62 [0.49–0.79]***	0.61 [0.48–0.78]***
*Pseudo R2*	0.08	0.08	0.08
*N*	3519	3422	3422

Note: RSOD i.e. risky single occasion drinking was defined as drinking 5+ standard drinks on one occasion at least once in a month

*p < 0.005,**p < 0.01,***p < 0.001

## Discussion

The present study has disentangled the relationship between socioeconomic status (SES), and risky single occasion drinking (RSOD), by quantifying the associations between a stable component of SES (education) as well as two variable components (employment and income) and drinking in a sample of the Danish general population. None of the components of SES were found associated with engaging in RSOD.

The fact that our results show education to be unrelated with reporting RSOD contradicts previous studies from other countries that have reported that lower education is associated with a disadvantageous risk profile such as heavy drinking [14, 27, 28]. Our results are on the other hand similar to those of Bloomfield et al. [29] who did not find significant differences in RSOD between distinct income and education groups in Denmark. But also as mentioned in the introduction, some studies have indeed found a positive relationship between SES and risky drinking in Denmark [30] and in other high income countries [31, 32]. With regard to other sociodemographic correlates of risky drinking, our findings agree with previous research including Danish studies [30, 33]; these include that being a woman, being in the older age groups versus the younger, living with children, and attending religious worship or ceremonies were found to be consistently protective against engaging in RSOD.

Regardless of how our results compare to previous studies, they do provide more recent and comprehensive examinations on how education and income relate to risky drinking in Denmark, and it seems that there are no particularly strong associations. Drinking may simply be normative, regardless of class: several studies, including qualitative investigations, have found daily drinking, binge drinking and intoxication-oriented weekend drinking to be the norm in many settings [34, 35]. Such a lifestyle does pervade all social classes to some extent in Denmark, and this may help explain why we found no correlation between SES and RSOD.

Some caveats are in order when interpreting the results. Firstly, although the response rate (64%) for our survey is higher than other recently conducted general population alcohol surveys both internationally [36, 37] and domestically [30, 33], we cannot rule out non-response bias. Although some studies have indicated that non-respondents could be either heavy drinkers or abstainers (see [38–40]) we could neither confirm this in the present study nor correct this possible bias. However, the extent of the bias might not be of major concern, as the estimated mean alcohol consumption in our study is comparable to official national estimates of per capita alcohol consumption [41]. Secondly, we used self-reported alcohol consumption, which might lead to under-reporting or over-reporting, as individuals tend to respond in a socially desirable way in order to conform to what they believe is appropriate or acceptable [42]. The tendency in alcohol survey research is toward underreporting, which, if present, would lead to conservative estimates (e.g., [43]). Thirdly, using personal income as measure of SES has been criticised since it does not include assets, savings, and properties [3]; unfortunately such data were not available for this study and therefore could not be explored.

Also, concerning our measures of SES, we defined employment as a dichotomous variable with all other statuses as being currently outside of the labour market, including being a homemaker or a student. However, in line with what is typically done on the literature, we focused only on people within the age span in which people are typically not studying or retired. In addition, it is quite uncommon to be a fulltime homemaker in Denmark.

## Conclusions

This study offers new data on the relationship between SES and RSOD. Our findings indicate that SES is unrelated to risky single occasion drinking in Denmark. To replicate and extend our findings, future research should include more detailed investigations based on long-term SES measures, such as life-time or permanent income, or income adjusted for household size as these additional measures may better capture the impact of social status on risky health behaviour than single-year measures can [44].
